# Triumeq Increases Excitability of Pyramidal Neurons in the Medial Prefrontal Cortex by Facilitating Voltage-Gated Ca^2+^ Channel Function

**DOI:** 10.3389/fphar.2020.617149

**Published:** 2021-01-28

**Authors:** Lihua Chen, Lena Al-Harthi, Xiu-Ti Hu

**Affiliations:** Department of Microbial Pathogens and Immunity, Rush University Medical Center, Chicago, IL, United States

**Keywords:** HIV-1, neurodegenerative disease, CART, neurotoxicity, hyperactivity, calcium dysregulation, voltage-gated calcium channel, electrophysiology

## Abstract

Combination antiretroviral therapy (cART) suppresses HIV-1 replication, improves immune function, and prolongs the life of people living with HIV (PLWH). However, cART also induces neurotoxicity that could complicate HIV-induced neurodegeneration while reduce its therapeutic efficacy in treating HIV/AIDS. Triumeq is a first-line cART regimen, which is co-formulated by three antiretroviral drugs (ARVs), lamivudine (3TC), abcavir (ABC), and dolutegravir (DTG). Little is known about potential side effects of ARVs on the brain (including those co-formulating Triumeq), and their mechanisms impacting neuronal activity. We assessed acute (*in vitro*) and chronic (*in vivo*) effects of Triumeq and co-formulating ARVs on pyramidal neurons in rat brain slices containing the medial prefrontal cortex (mPFC) using patch-clamp recording approaches. We found that acute Triumeq or 3TC *in vitro* significantly increased firing of mPFC neurons in a concentration- and time-dependent manner. This neuronal hyperactivity was associated with enhanced Ca^2+^ influx through voltage-gated Ca^2+^ channels (VGCCs). Additionally, chronic treatment with Triumeq *in vivo* for 4 weeks (4 wks) also significantly increased firing and Ca^2+^ influx via VGCCs in mPFC neurons, which was not shown after 2 wks treatment. Such mPFC neuronal hyperexcitability was not found after 4 weeks treatments of individual ARVs. Further, chronic Triumeq exposure *in vivo* significantly enhanced mRNA expression of low voltage-activated (LVA) L-type Ca^2+^ channels (Ca_v_1.3 L-channels), while changes in high voltage-activated (HVA) Cav1.2 L-channels were not observed. Collectively, these novel findings demonstrate that chronic cART induces hyperexcitability of mPFC pyramidal neurons by abnormally promoting VGCC overactivation/overexpression of VGCCs (including, but may not limited to, LVA-Ca_v_1.3 L-channels), which could complicate HIV-induced neurotoxicity, and ultimately may contribute to HIV-associated neurocognitive disorders (HAND) in PLWH. Determining additional target(s) of cART in mPFC pyramidal neurons may help to improve the therapeutic strategies by minimizing the side effects of cART for treating HIV/AIDS.

## Introduction

Combination antiretroviral therapy (cART) effectively suppresses HIV replication, improves immune function, and prolongs life of people living with HIV (PLWH), which transformed HIV infection from a fatal disease to a lifelong chronic, but manageable disease. However, the prevalence of HIV-associated neurocognitive disorders (HAND) occurs in up to ∼50% of PLWH in the era of cART (Antinori et al., 2007; Tozzi et al., 2007; McArthur et al., 2010). A growing body of evidence suggests that cART may be a contributing factor to the neurodegenerative HAND, likely by altering the function of the brain regions that regulate cognition (Heaton et al., 2010; Robertson et al., 2010; Simioni et al., 2010). It is unknown if this high prevalence of HAND is due to the legacy effects in the pre-cART era, low level of HIV below the limit of detection of sensitive assays, or the side effects of cART that could complicate HIV-induced neurotoxicity, or both (Kranick and Nath, 2012; Manji et al., 2013; Underwood et al., 2015), especially during aging (Brew et al., 2009; Cysique and Brew, 2009; Vance et al., 2011; Jiang et al., 2016; Clifford et al., 2017). Therefore, understanding how and to what extent chronic cART affects the functional activity of neurons in the key brain regions that regulate neurocognition in PLWH warrants a thorough investigation.

The medial prefrontal cortex (mPFC) plays a critical role in regulating neurocognition; but it is profoundly altered by HIV-1 (Ferris et al., 2008; Hu, 2016). HIV-1 induces hyperactivity of glutamatergic pyramidal neurons in the mPFC by causing overactivation of voltage-gated Ca^2+^ channels (VGCCs) (Khodr et al., 2016; 2018; Napier et al., 2014; Wayman et al., 2015; 2016; 2012), in addition to glutamate and NMDA receptor (NMDAR) dysfunction (Gonzalez-Scarano and Martin-Garcia, 2005; King et al., 2006). Such Ca^2+^ dysregulation-mediated neurotoxicity resulting from NMDAR/VGCC dysfunction may contribute to the underlying mechanism of HAND (Hu, 2016). Other studies also suggest a negative impact of antiretroviral drugs (ARVs) on cortical neurons that may worsen HIV-induced neurotoxicity and HIV/AIDS-associated neurodegenerative consequences (Akay et al., 2011; Birbeck et al., 2012; Robertson et al., 2012; Romo et al., 2012; Manji et al., 2013; Akay et al., 2014; Jensen et al., 2015). Whether, how, and to what extent chronic cART alters neuronal activity in the mPFC, or in other brain regions that also regulate neurocognition, and therefore complicates HIV neurotoxicity, is not known.

Triumeq is a first-line cART regimen recommended by the World Health Organization (WHO) for treating HIV/AIDS (Walmsley et al., 2013; Calcagno et al., 2014; Greig and Deeks, 2015; Nwogu et al., 2016). It is a single-tablet co-formulated by three ARVs: lamivudine (3TC), abcavir (ABC) and dolutegravir (DTG) (Walmsley et al., 2013). 3TC and ABC are nucleoside reverse transcriptase inhibitor (NRTI), while DTG is an integrase strand transfer inhibitor (INSTI). Together, they effectively inhibit HIV replication. However, despite the required therapeutic effects, many ARVs also induce neurotoxicity, which could exacerbate HIV-induced neurodegeneration and brain dysfunction (Gonzalez et al., 2020). Clinical studies report that chronic treatments of ABC, DTG, efavirenz, and some other ARVs are also associated with neuropsychiatric disorders (Maxwell et al., 1988; Blanch et al., 2001; Colebunders et al., 2002; Wise et al., 2002; Mengato et al., 2020). Other studies show that some side effects of 3TC and DTG disappear when HIV^+^ patients stop taking ARVs (Zamora et al., 2019). Moreover, a recent clinical study also reveals that HIV^+^ women with consistent viral suppression following continuous cART show significantly more cognitive deficits than HIV^−^ or HIV^+^ women without chronic cART (Rubin et al., 2017).

Pre-clinical studies using lab animals also reveal ARVs-induced neurotoxicity in the brain, including, but not limited to, loss of the dendritic processes, cytoplasmic shrinkage, mitochondrial dysfunction, and death of prefrontal cortical neurons in SIV^+^ macaques and rats after chronic cART *in vivo* (Robertson et al., 2012; Akay et al., 2014). Chronic treatment with tenovovir, 3TC, and efavirenz also disturbs activity of hippocampal pyramidal neurons and memory impairments in rats (Akang, 2019), while acute ARV treatment reduces bioenergetic function in nerve terminals isolated from the striatum (Stauch et al., 2017). It is worth noting that 1) the prefrontal cortex, hippocampus, and striatum are the key regulators of cognition; but they are also most susceptible and vulnerable to HIV (Ferris et al., 2008; Manji et al., 2013; Hu, 2016); and (ii) neurotoxicity induced by ARVs appears to be associated with dysregulation of neuronal Ca^2+^ homeostasis (i.e., excessive intracellular free calcium, [Ca^2+^]_in_) (Robertson et al., 2012; Romo et al., 2012; Akay et al., 2014; Apostolova et al., 2015; Underwood et al., 2015; Vivithanaporn et al., 2016), similar to that induced by HIV (Wayman et al., 2012, 2015, 2016; Napier et al., 2014; Khodr et al., 2016, 2018; Chen et al., 2019). Collectively, these studies suggest that the site effects of cART disturb neuronal activity in the brain regions that regulate neurocognition, and therefore indicate the necessity to elucidate the mechanism by which ARVs disrupt the brain function. Understanding such mechanism could help us to improve the therapeutic strategies for treating HIV/AIDS by minimizing the side effects of cART through pharmacological intervene.

In this study, we assessed the impact of Triumeq, both acutely (*in vitro*) and chronically (*in vivo*), and the three individual ARVs (3TC, ABC, and DTG) co-formulating it, on the functional activity of mPFC pyramidal neurons in rat brain slices. We also defined the mechanism (i.e., overactivation of VGCCs) by which Triumeq induces abnormal neuronal hyperactivity that may complicate HIV-induced neurotoxicity in the mPFC.

## Materials and Methods

### Animals

Male F344 rats (3–4 weeks old, wk) were purchased from Charles River (Wilmington, MA) and group-housed at Rush University Medical Center animal facility on a 12-hour light/dark cycle. Food and water were available *ad libitum*. Animal care and use procedures were conducted with the Institutional Animal Care and Use Committee, and in accordance with NIH, USDA and institutional guidelines.

### Whole-Cell Current-Clamp Recording

Brain slices of rats were prepared, and then evoked action potential (firing) and VGCC activity were assessed among pyramidal neurons from the layer V-VI of the mPFC as described in our previous studies (Nasif et al., 2005a; Wayman et al., 2005b; Napier et al., 2014; Wayman et al., 2015; Wayman et al., 2016, Chen et al., 2019; Khodr et al., 2018).

To access the normal action potential, glass electrodes (4–6 MΩ) were filled with an internal solution (in mM: 120 K-gluconate, 10 HEPES, 20 KCl, 2 MgCl_2_, 3 Na_2_ATP, 0.3 NaGTP, and 0.1 EGTA; pH: 7.3–7.35; with 280–285 mOsm). All neurons met the criteria of a resting membrane potential (RMP) at least −60 mV and action potential (AP) amplitude ≥60 mV. The recording protocol included a series of 500 ms current pulses with the intensity ranged from 0 to +300 pA with a 25-pA.

VGCC activity was accessed after ∼10 min perfusion of aCSF containing selective blockers for voltage-sensitive Na^+^ channels (tetrodotoxin, TTX, 0.5 µM), K^+^ channels (tetraethylammonium, TEA, 20 mM; Sigma), NMDA/AMPA receptors (kynurenic acid, 3 mM), and GABA_A_ receptors (GABA_A_R; picrotoxin, 100 μM). The internal solution used in the recording of VGCC activity consisted of (in mM) 140 Cs-gluconate, 10 HEPES, 2 MgCl_2_, 3 Na_2_ATP and 0.3 NaGTP (pH: 7.3–7.35; with 280–285 mOsm). *V*_m_ was held at ∼−68 mV (around average RMP). 40 ms depolarizing rheobase currents were determined and used to elicit Ca^2+^ plateau potentials (reflecting Ca^2+^ influx through VGCCs).

### Drug Application

For 3TC study *in vitro*, 3TC (from NIH AIDS reagent program) was dissolved in ddH_2_O to make 10 mg/ml or 100 mg/ml stock solution. For Triumeq study *in vitro*, 1-fold (1×) Triumeq contained 1 µM (300 ng/ml) ABC, 1.3 µM (300 ng/ml) 3TC, and 50 nM (20 ng/ml) DTG dissolved in ddH_2_O, ddH_2_O and DMSO (vehicle), respectively. Such drug concentrations are equivalent or comparable to the levels of these ARVs reported in the CSF of cART-treated HIV^+^ patients (Calcagno et al., 2014; Van den Hof et al., 2018). To access the drug(s) effects on firing and VGCC activity, at least 1000x higher concentrated stock solution of the compound(s) were diluted in the aCSF to make the final concentration. Recording was performed after ≥10 min perfusion of each concentration from low to higher concentration using a continuous perfusion system.

During *in vivo* study, ABC and 3TC (Sigma, St. Louis, MO) were dissolved in saline (SAL) to make 12 mg/ml and 6 mg/ml stock solution, respectively. DTG were first dissolved in 100% DMSO and then diluted to 70% DMSO with SAL (vehicle). The final DTG stock concentration was 1 mg/ml. During chronic experiment, rats received one daily s. c. injection of each compound based on their body weight. As a result, animals received a combined daily dosage of 12 mg/kg ABC, 6 mg/kg 3TC, and 1 mg/kg DTG. The combined dosage was similar to the Triumeq pill taken by PLWH. All drugs were purchased from Sigma unless specified.

### Quantitative Real-Time PCR

Anesthetized rats were perfused transcardially with ice-cold saline. The brain was removed and dissected mPFC tissues were stored at −80°C for RNA extraction. Total RNA was extracted using a miRNeasy Mini kit (Qiagen Inc., Germantown, MD). DNA was removed using the RNase-free DNase set (Qiagen Inc.). mRNA level was measured on an Applied Biosystems (Foster City, CA) QuantStudio 7 Flex real-time PCR system using the Power SYBR® Green RNA-to-C_T_™ 1-Step kit (Applied Biosystems). The 20 μL reaction mixture contained 25 ng of total RNA, 10 μL of 2X Power SYBR® Green RT-PCR Mix, 0.14 μL of RT enzyme mix, and 0.2 μL of each primer (10 μM forward β-actin, or 20 μM forward *CACNA1c* or *CACNA1d* and 20 μM reverse β-actin, *CACNA1C* or *CACNA1D*). Primers used are as follows: rat β-actin (forward primer: ccg​cga​gta​caa​cct​tct​tgc, reverse primer: ata​tcg​tca​tcc​atg​gcg​aac​tgg); rat *CACNA1C* (forward primer: ggc​atc​acc​aac​ttc​gac​a, reverse primer: tac​acc​cag​ggc​aac​tca​ta); rat *CACNA1D* (forward primer: gag​agg​agg​gca​aac​gaa​aca, reverse primer: tct​ttt​cca​cca​gca​cca​gag​a). Reactions were incubated at 48°C for 30 min; 95°C for 10 min, then 40 cycles of 95°C for 15 s and 60°C for 1 min, followed by a dissociation curve in order to confirm primer specificity. The mRNA levels shown as fold change (2^−ΔΔCt^) were normalized to β-actin Ct and to vehicle (4 wk).

### Statistical Analysis

Prism (GraphPad Software Inc., La Jolla, CA) and SigmaPlot (Systat Software Inc., San Jose, CA) were used to analyze data. One-way ANOVA with repeated measures (rm) was used to analyze the membrane properties and VGCC activity, followed by Dunnett’s *post-hoc* test. Two-way rm ANOVA was used to analyze the firing frequency followed by Newman-Keuls *post-hoc* test. Statistical significance was set at *p* ≤ 0.05. Student *t*-test was used to analyze the mRNA level of L-type Ca^2+^ channels. Data was presented as mean ± standard error. Outlier was identified as more than 2× of the standard deviation from the mean and was excluded from analysis.

## Result

### Acute Lamivudine Treatment *in vitro* Increases Firing and Voltage-Gated Ca2+ Channel Activity of Medial Prefrontal Cortex Pyramidal Neurons in a Dose-dependent Manner

**Figure 1:**
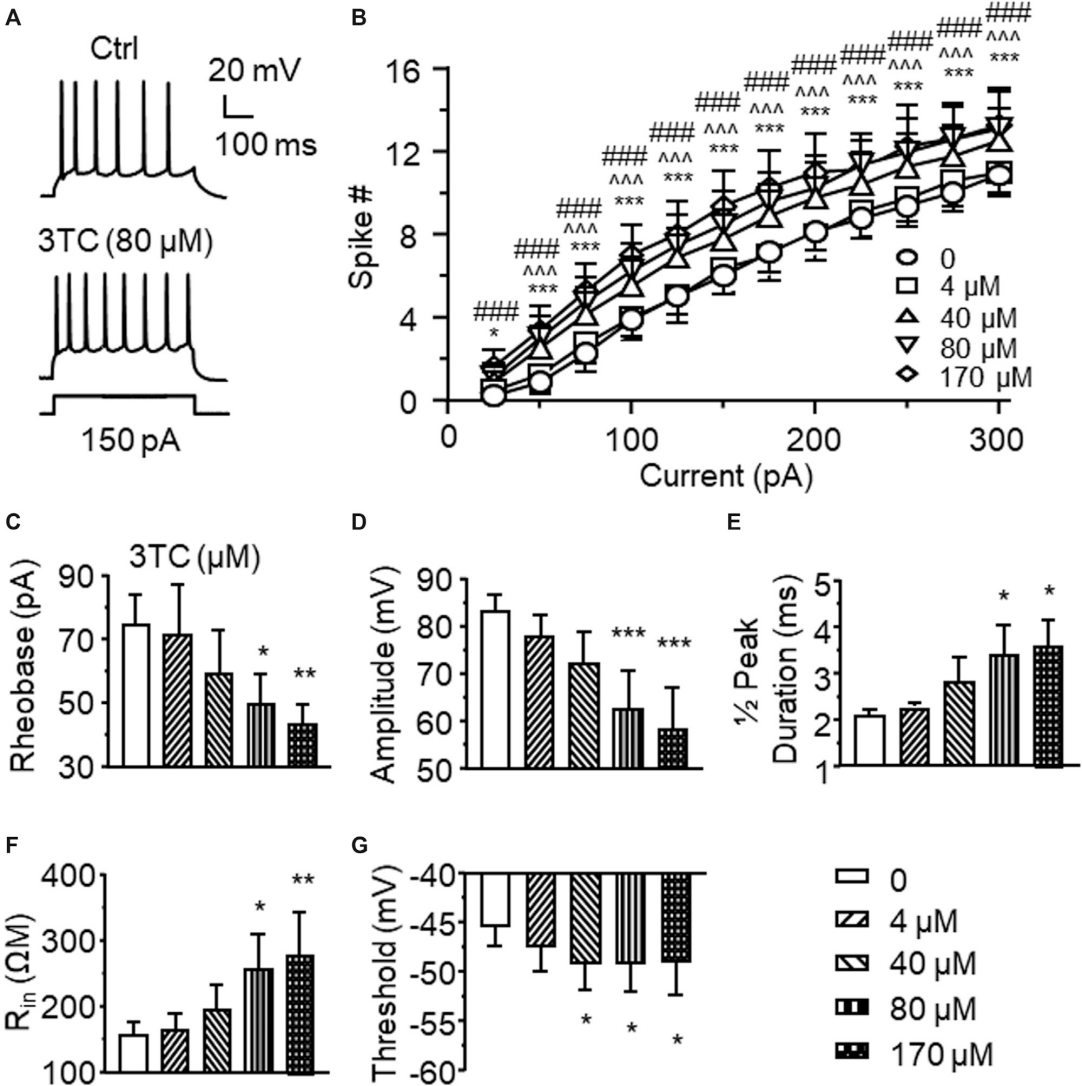
Acute treatment of lamivudine (3TC) *in vitro* increases firing of mPFC pyramidal neurons **(A)** Sample traces showing that a moderate depolarizing current pulse (150pA, which mimicked physiological excitatory inputs) evoked more action potentials in a 3TC-treated neuron compared to one without 3TC treatment **(B)** The current-spike relationships indicate a significant increase in the firing frequency of neurons treated with 3TC in a concentration-dependent manner (40–170 µM) compared to those without this ARV (n = 8/ea. ***p < 0.001 between 0 vs 44 µM; ^  ^^^*p* < 0.05 or 0.001 between 0 and 80 µM; ###*p* < 0.001 between 0 and 170 µM). 3TC-Associated with increased firing, higher concentrations of 3TC also altered the membrane properties by reducing the rheobase **(C)** and peak amplitude **(D)**, increasing the ½ peak duration **(E)** and inward resistance R_in_ **(F)**, while decreasing the threshold **(G)** (n = 8/ea. *^,^**^,^***p < 0.05, 0.01 or 0.001).

To determine the impact of Triumeq on mPFC neuronal activity, we first assessed the acute effects of lamivudine (a.k.a. 3TC, one of the ARVs that co-formulates Triumeq) on firing of mPFC pyramidal neurons evoked by depolarizing current pulses (25–400 pA). We found that 3TC at the concentrations of 40, 80 and 170 µM, though not 4 µM, significantly increased neuron’s firing (n = 8/ea. 3TC effect: F_(4,28)_ = 10.77, *p* < 0.001; Current effect: F_(11,77)_ = 123.3, *p* < 0.001; Interaction: F_(44,308)_ = 1.725, *p* = 0.004; Figures 1A,B). Certain membrane properties were altered in association with the increased firing, which were also in a concentration-dependent manner. For example, the rheobase (the minimal current required for evoking firing, n = 8/each group (ea), F_(4,28)_ = 5.174, *p* = 0.003) and peak amplitude (n = 8/ea, F_(4,28)_ = 11.512, *p*<0.001) were significantly reduced by 3TC at higher doses (80 and 170 µM) (Figures 1C,D), the half-peak duration (n = 8/ea, F_(4,28)_ = 4.080, *p* = 0.01) and inward resistance (R_in_, n = 8/ea, F_(4,28)_ = 4.665, *p* = 0.005) were significantly increased by 80 and 170 µM 3TC (Figures 1E,F), while the threshold was reduced by 40, 80 and 170 µM 3TC (n = 8/ea, F_(4,28)_ = 2.989, *p* = 0.036; Figure 1G). All these alterations indicate an increased membrane excitability among these neurons.

**Figure 2:**
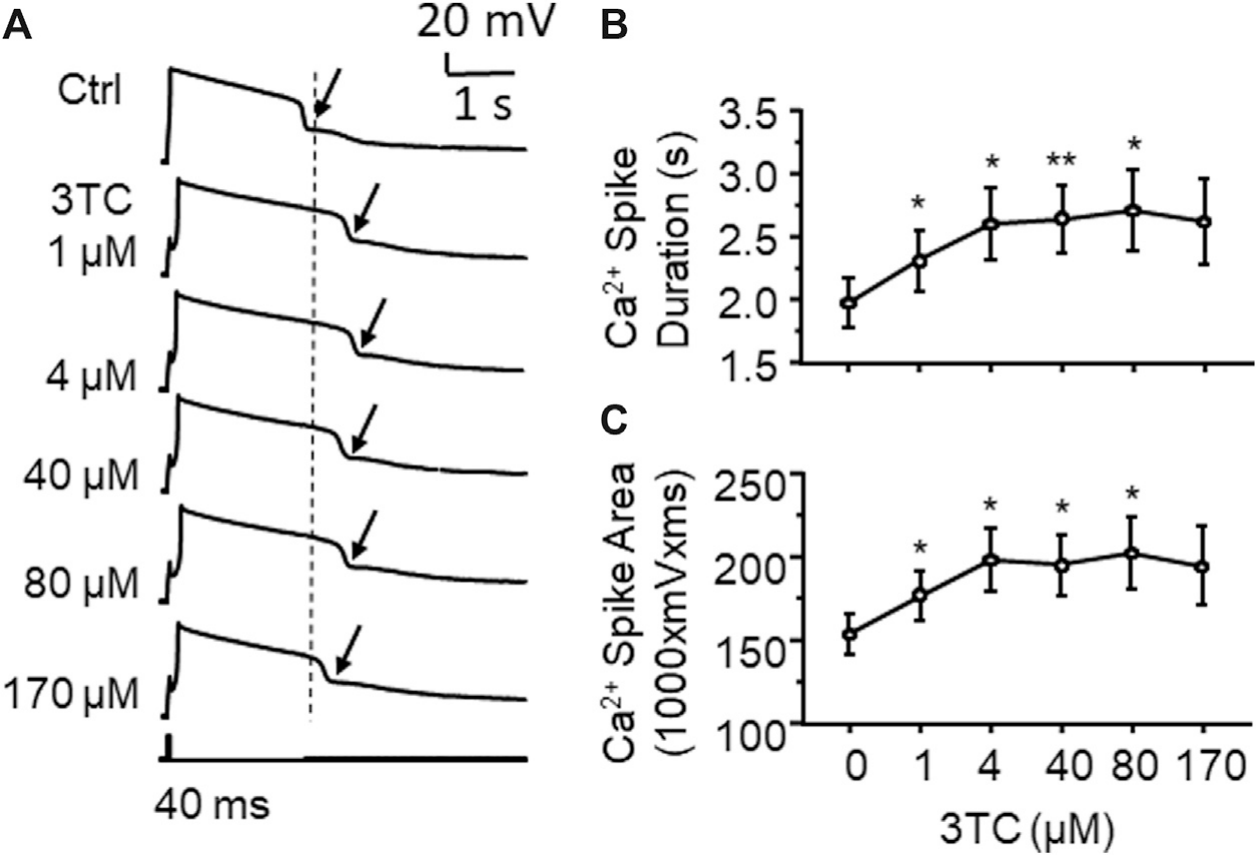
3TC enhances Ca^2+^ influx through overactive VGCCs **(A)** Sample traces showing that acute exposure to 3TC *in vitro* induced a prolongation of Ca^2+^ spikes in mPFC neurons (pointed by the arrows), indicating an increased functional activity of VGCCs in mPFC neurons **(B)** The acute effect of 3TC on Ca^2+^ spikes was also concentration-dependent, showing that the duration of Ca^2+^ spike was gradually increased at lower concentrations (1–80 µM), but begun to decrease in response to a high concentration (170 µM) (n = 9/ea. *^,^**p < 0.05 or 0.01).

VGCCs are one of the key regulators in mediating neuronal excitability. To determine if alterations in Ca^2+^ channel activity contribute to 3TC-induced increase of firing, voltage-sensitive Ca^2+^ spikes (reflecting Ca^2+^ influx *via* activated VGCCs) were evaluated with blockade of voltage-gated Na^+^/K^+^ channels, as well as glutamate and GABA-mediated excitatory and inhibitory inputs, respectively. We found that voltage-sensitive Ca^2+^ influx through VGCCs in mPFC pyramidal neurons was significantly enhanced by 3TC in a concentration-dependent manner (1, 4, 40 and 80 µM), evidenced by increased Ca^2+^ spike duration (n = 9/ea. F_(5,40)_ = 5.052, *p* = 0.001) and area (n = 9/ea. F_(5,40)_ = 4.068, *p* = 0.004) (Figure 2A). But 170 µM 3TC begun to reduce the previously prolonged Ca^2+^ spike (Figures 2B,C), likely due to overactivation-induced inactivation of VGCCs. The lowest concentration (1 µM) used here was equivalent to the reported level of 3TC in the CSFs of HIV^+^ patients taking this ARV, while the higher concentrations (4∼40 µM) were comparable to the plasma levels of this ARV detected in HIV^+^ individuals (Johnson et al., 1999; Van den Hof et al., 2018). The highest concentrations of 3TC (80 and 170) were used to assess potential neurotoxic effect of this ARV on dysregulating neuronal Ca^2+^ homeostasis mediated by VGCCs, as done in our previous studies (Napier et al., 2014; Wayman et al., 2015; Wayman et al., 2016). These results reveal a 3TC-induced increase in VGCC activity and Ca^2+^ influx, which contributes to increase firing.

### Acute Triumeq Exposure *in vitro* Increases Firing of Medial Prefrontal Cortex Neurons and Voltage-Sensitive Ca^2+^ Influx *in vitro* in a Dose-Dependent Manner

**Figure 3:**
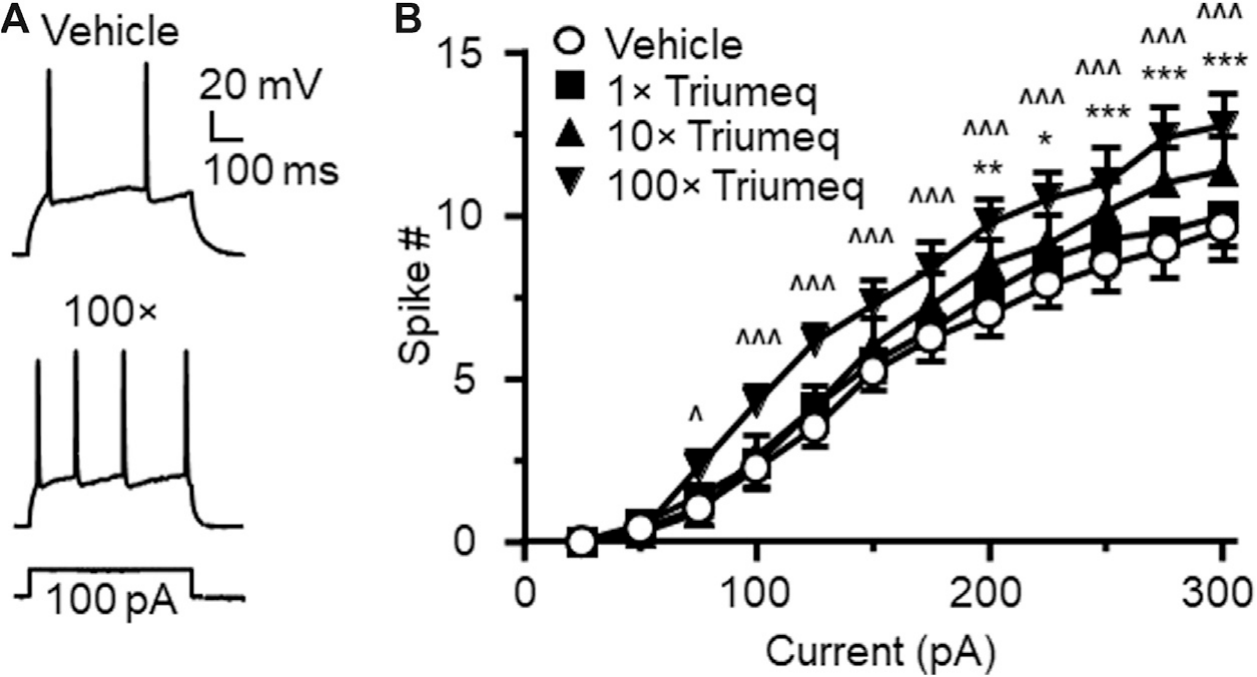
Acute Triumeq treatment *in vitro* also increases firing of mPFC pyramidal neurons in a dose-dependent manner **(A)** Sample traces showing the firing numbers evoked by a moderate depolarizing current (150 pA) in mPFC neurons with or without Triumeq in the bath **(B)** The current-spike relationships indicate a significant increase in mPFC neuronal firing in response to 10× or 100×, but not 1×, of acute Triumeq compared to vehicle-treated controls (n = 8/ea. Triumeq effect: *F*_3,21_ = 5.986, *p* = 0.004; current effect: *F*_11,77_ = 93.88, *p* < 0.001; interaction: *F*_33,231_ = 3.697, *p* < 0.001. *post-hoc* test: *^,^**^,^***p < 0.05, 0.01 or 0.001 between 0 vs 10× Triumeq; ^^,^^^^*p* < 0.05 or 0.001 between 0 vs 100× Triumeq). 1× Triumeq (containing 0.3 μg/ml ABC, 0.3 μg/ml 3TC, and 20 ng/ml DTG) equals to the concentrations of these ARVs found in the CSF of PLWH on Triumeq. 10× and 100× Triumeq refers to 10-fold and 100-fold of which, respectively.

**Figure 4:**
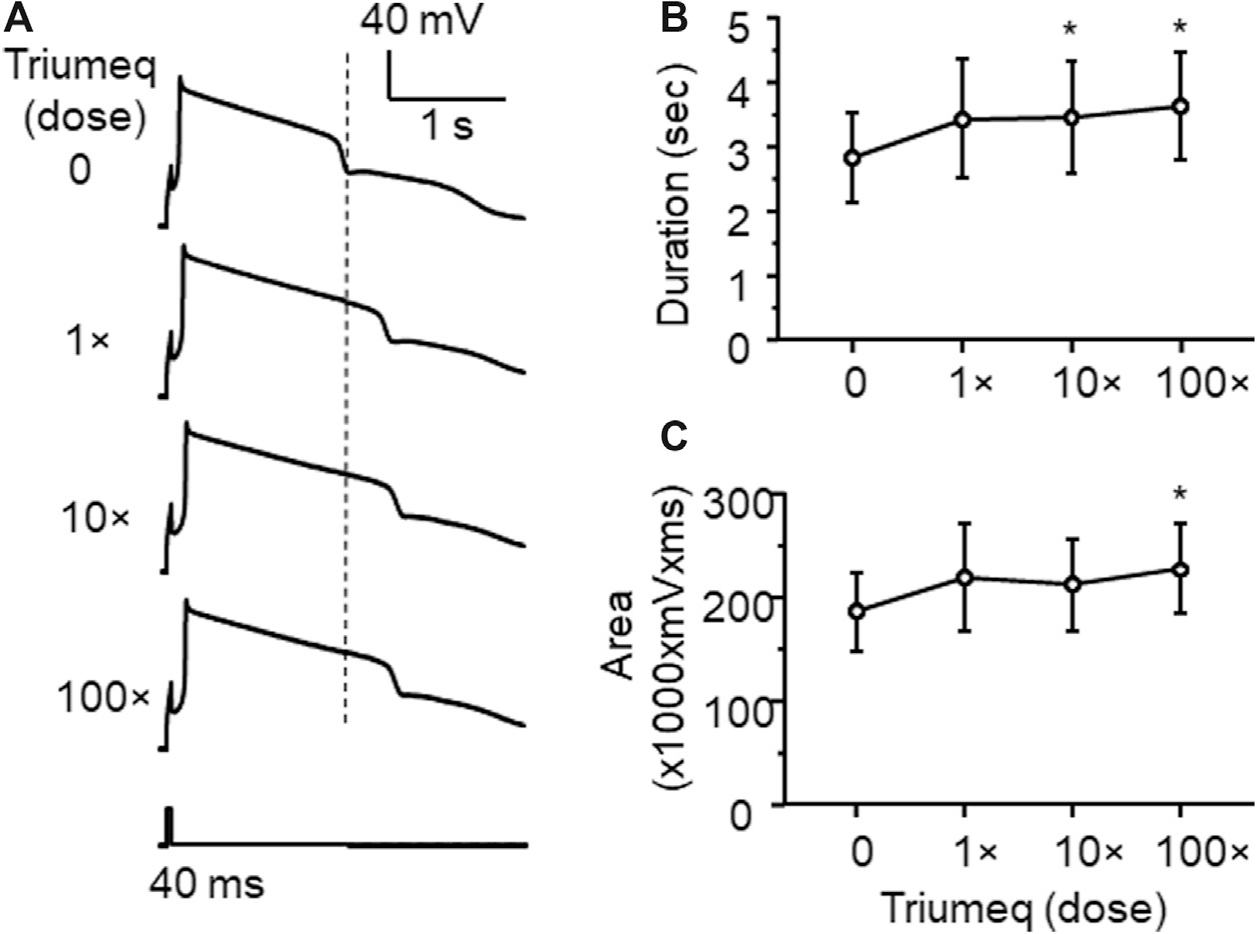
Acute Triumeq treatment also increases VGCC activity in mPFC pyramidal neurons in a dose-dependent manner **(A)** Sample traces showing the acute effects of Triumeq (1×, 10×, and 100×) on evoked Ca^2+^ spikes **(B–C)** High concentrations of Triumeq *in vitro* significantly increased the duration **(B)** (n = 8/ea. *F*_3,21_ = 7.089, *p* = 0.002) and the area of Ca^2+^ spikes **(C)** (n = 8/ea. *F*_3,21_ = 4.201, *p* = 0.018). *p<0.05 compared to the baseline prior to acute exposure to Triumeq.

To access the acute effects of Triumeq on functional activity of mPFC neurons, we evaluated the firing frequency of neurons in response to depolarizing current pulses (25–400 pA) after ≥10 min perfusion with different concentrations of Triumeq in the bath. Similar to our 3TC study, we assessed the acute effects of Triumeq on mPFC neurons using different concentrations. The low concentration (1×) was the same as ×that found in the CSFs of HIV^+^ patients on this cART, while higher concentrations (up to 100×) were comparable to that found in the plasma of HIV^+^ on this cART since different ARVs have different plasma:CSF ratios (e.g., the plasma level of 3TC was about twice of that in CSF, and the plasma level of DTG was 236× higher than it in CSF) (Letendre et al., 2014). We found that the 1× Triumeq induced no alteration in firing; but at the concentrations that were 10- and 100-fold (10× and 100×, respectively) higher than the levels found in the CSFs of PLWH, Triumeq significantly increased firing of mPFC neurons compared to vehicle-treated controls (n = 8 neurons in 8 rats per group data; Triumeq effect: *F*_(3,21)_ = 5.986, *p* = 0.004; current effect: F_(11,77)_ = 93.88, *p* < 0.001; interaction: *F*_(33,231)_ = 3.697, *p* < 0.001; Figure 3A,B). Moreover, we also assessed the effects of Triumeq *in vitro* on the functional activity of VGCCs. We found that Ca^2+^ spike was significantly increased by acute Triumeq, also in a dose-dependent manner (Figure 4A). For example, the duration and/or area were significantly prolonged and enlarged by higher concentrations of Triumeq (10× and 100×, both *p < 0.05), but not by 1× Triumeq, in the bath (Figures 4B,C).

### Chronic Triumeq Treatment *in vivo* Increases Firing of Medial Prefrontal Cortex Neurons

**Figure 5:**
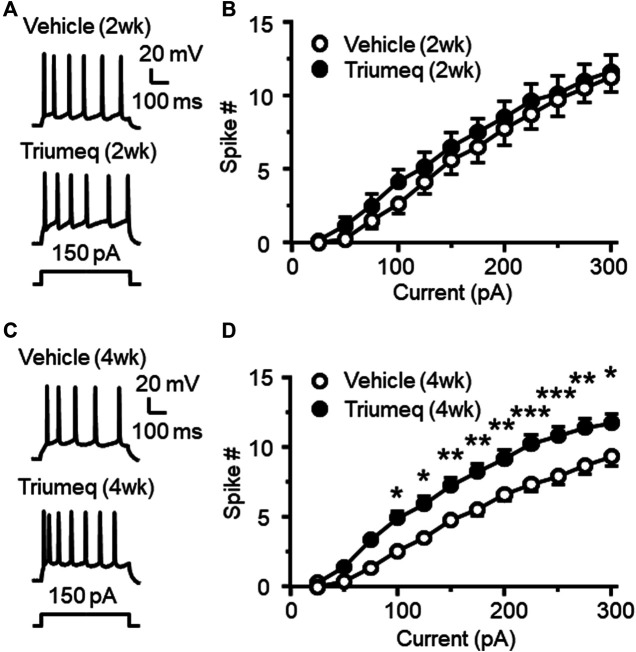
Chronic, but not subchronic, treatment of Triumeq *in vivo* significantly increases firing of mPFC pyramidal neurons **(A)** Sample traces showing firing of mPFC neurons evoked by *V*_m_ depolarization after a 2 wks subchronic vehicle **(upper panel)** or Triumeq **(lower panel)** treatment *in vivo* **(B)** The current-spike relationships indicate that there was no significant difference in the firing between mPFC neurons from 2 wks Triumeq-pretreated rats and those from 2 wks vehicle-pretreated controls (n = 8/ea. Triumeq effect: *F*_1,14_ = 0.424, *p* = 0.426; current effect: *F*_11,154_ = 187.8, *p* < 0.001; interaction: *F*_11,154_ = 0.418, *p* = 0.947) **(C)** Sample traces showing the changes in firing evoked by *V*_m_ depolarization in mPFC neurons after 4 wks of vehicle **(upper panel)** or Triumeq **(lower panel)** treatment *in vivo* **(D)** The current-spike relationships indicate a significant increase in firing of mPFC neurons in rats after 4 wks Triumeq pretreatment compared to those in 4 wks vehicle-pretreated rats (14/ea. Triumeq effect: *F*_1,26_ = 12.59, *p* = 0.002; current effect: *F*_11,286_ = 398.9, *p* < 0.001; interaction: *F*_11,286_ = 4.851, *p* < 0.001; *post-hoc* test, *^,^**^,^***p < 0.05, 0.01 or 0.001).

To access the chronic effects of Triumeq *in vivo* on neuronal excitability, we evaluated the firing frequency of mPFC pyramidal neurons in rats after 2 wks (subchronic) or 4 wks (chronic) once daily s. c. injections of Triumeq. There was no significant difference in evoked firing between mPFC neurons from rats pretreated with vehicle and Triumeq for 2 wks (Figures 5A,B); but firing was significantly increased following chronic Triumeq treatment for 4 wks (*,**,***p < 0.05, 0.01, and 0.001, Figures 5C,D). These findings suggest that chronic exposure to Triumeq *in vivo* for 4 wks significantly increases mPFC neuronal excitability, while earlier changes in neuronal excitability induced by acute or subchronic (2 wks) treatment of Triumeq might be compensated by a negative-feedback mechanism in these neurons.

The increased firing following 4 wks treatment of Triumeq *in vivo* was associated with alterations in some membrane properties (Table 1). For example, the rheobase was significantly decreased in mPFC neurons from 4 wks Triumeq-pretreated rats (n = 15 neurons in 4 rats per group data; *t*_28_ = 2.294, *p* = 0.0295), while the ½ peak duration was significantly increased after 4 wks Triumeq-pretreatment compared to vehicle-pretreated controls (Vehicle vs Triumeq-4 wks: n = 14 vs. 15 neurons in 4 rats; *t*_27_ = 2.29, *p* = 0.0301). The reduced rheobase suggests an increased mPFC neuronal excitability, which is in agreement with 4 wks Triumeq-induced increase of firing. The ½ peak duration is partly mediated by VGCCs, and therefore, a prolonged ½ peak duration following 4 wks Triumeq-pretreatment suggests increased activity of VGCCs.

**TABLE 1:** Chronic Triumeq treatment significantly alters some membrane properties in mPFC pyramidal neurons.

	Vehicle (4 wks)	Triumeq (4 wks)
RMP (mV)	−64.4 ± 1.1	−62.4 ± 0.7
R_in_ (MΩ)	208.5 ± 14.0	242.8 ± 14.9
Rheobase (pA)	71.7 ± 5.9	53.3 ± 5.4*
Threshold (mV)	−40.6 ± 1.2	−42.7 ± 0.8
Amplitude (mV)	79.1 ± 2.4	71.7 ± 3.3
½ peak duration (ms)	2.2 ± 0.1	2.5 ± 0.1*
Time constant (ms)	39.1 ± 4.0	34.5 ± 2.4
AHP (mV)	11.6 ± 1.0	9.4 ± 1.2

*t*-test: *p < 0.5.

### Chronic Triumeq Treatment Enhances Ca^2+^ Influx via Voltage-Gated Ca2+ Channel

**Figure 6:**
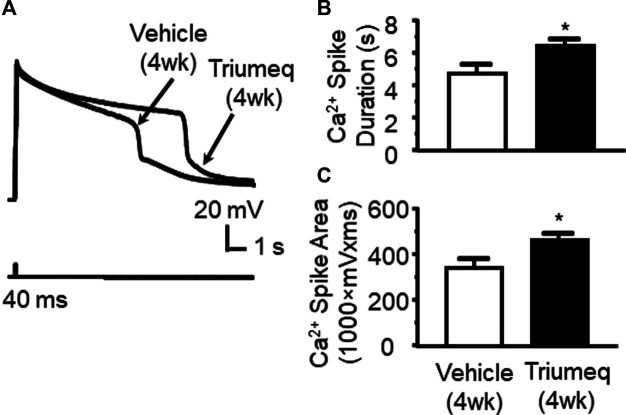
Chronic Triumeq treatment *in vivo* for 4 wks increases Ca^2+^ influx via VGCCs in mPFC pyramidal neurons compared to those from vehicle-pretreated controls **(A)** Sample traces showing Ca^2+^ spikes evoked by *V*_m_ depolarization (indicating Ca^2+^ influx *via* VGCCs) in mPFC neurons from a vehicle-pretreated rat compared to a Triumeq-pretreated rat **(B)–(C)** The bar graphs indicate that the duration **(B)** and area **(C)** of Ca^2+^ spikes were significantly prolonged and enlarged, respectively, after a 4 wks Triumeq pretreatment (the duration: vehicle vs. Triumeq: n = 8 vs. 11; *t*_17_ = 2.141, *p* = 0.047; and the area: vehicle vs. Triumeq: n = 8 vs. 11; *t*_17_ = 2.272, *p* = 0.036).

To determine the chronic effects of Triumeq *in vivo* on VGCC activity, we assessed Ca^2+^ spikes evoked in mPFC pyramidal neurons of rats after 4 wks Triumeq or vehicle pretreatment. We found that Ca^2+^ spike was significantly prolonged by chronic Triumeq treatment for 4 wks (Figure 6A). Both the duration (*p* < 0.05; Figure 6B) and spike area (*p* < 0.05; Figure 6C) were significantly increased in mPFC neurons from rats pretreated with Triumeq for 4 wks compared to those from vehicle-pretreated rats. These findings indicate that chronic Triumeq treatment increases VGCC activity in mPFC neurons; and such change in intracellular Ca^2+^ ([Ca^2+^]_in_) could contribute to the abnormal increase of firing.

### Chronic Triumeq Treatment *in vivo* Increases the mRNA Expression of Ca_v_1.3 L-Channels

**Figure 7:**
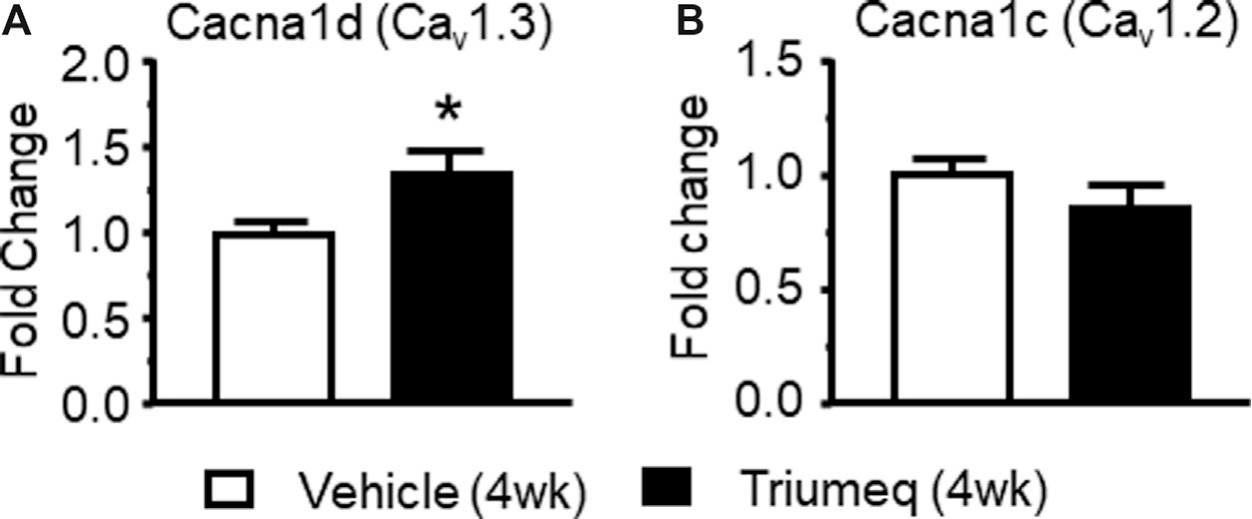
Chronic Triumeq treatment *in vivo* for 4 wk significantly increases the mRNA expression of Ca_v_1.3, but not Cav1.2, L-channels in the mPFC **(A)** The mRNA level of the Ca_v_1.3 L-channel (Cacna1d gene) was significantly increased in the mPFC following 4 wks treatment of Triumeq compared to chronic treatment of vehicle (n = 8 rat/ea: *t*_14_ = 2.161, *p* = 0.0485) **(B)** There was no significant change in the mRNA level of Ca_v_1.2 (Cacna1c gene) L-channels in the mPFC after 4 wks treatment of Triumeq (n = 8 rat/ea: *t*_14_ = 1.249, *p* = 0.232).

There are different subtypes of VGCCs (Brini et al., 2014), including the L-type (L-channel). In the brain, L-channels consist of a high voltage-activated (HVA-Ca_v_1.2) and a low voltage-activated (LVA-Ca_v_1.3) form (Lipscombe, 2002). To better understand which subtype of L-channels contributes to the Triumeq-induced increase of Ca^2+^ spikes, we assessed the mRNA expression levels of Ca_v_1.2 (a LVA L-channel) and Ca_v_1.3 (a HVA L-channel) using qRT-PCR. We found that the Ca_v_1.3 mRNA level was significantly increased in the mPFC from rats after chronic Triumeq pretreatment compared to vehicle-pretreatment rats (*p < 0.05; Figure 7A), while the Ca_v_1.2 was not changes (Figure 7B). These results demonstrate that LVA-Ca_v_1.3 L-channels mediate Triumeq-induced increase of mPFC neuronal excitability.

### Chronic Treatment of Individual Antiretroviral Drugs Alone Does Not Alter Firing of Medial Prefrontal Cortex Neurons

**Figure 8:**
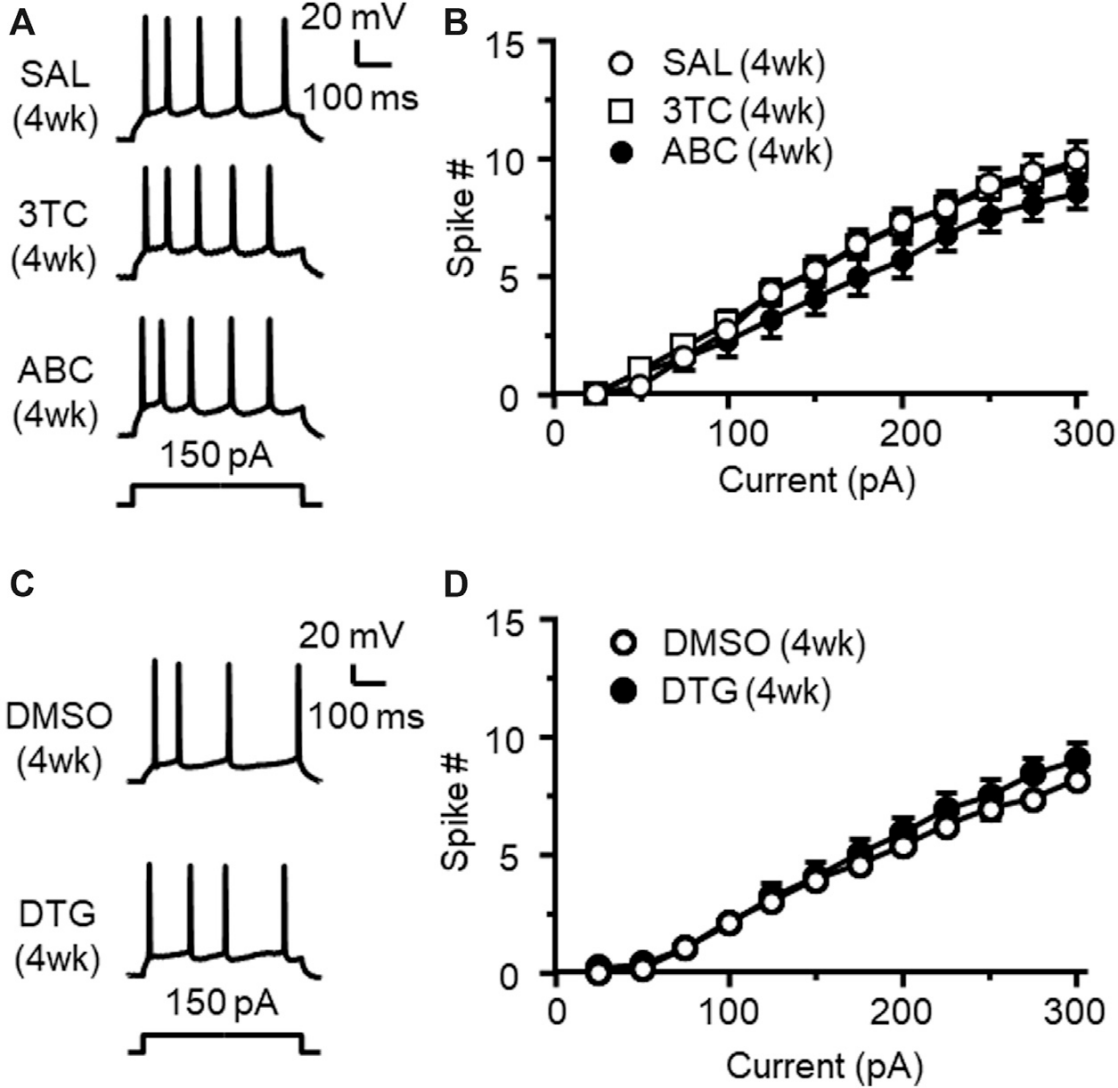
Chronic treatment of individual 3TC, ABC, or DTG alone *in vivo* for 4 wks does not alter firing of mPFC pyramidal neurons **(A)** Samples traces showing firing in response to *V*_m_ hyperpolarization in mPFC neurons following 4 wks chronic treatment of SAL (upper panel), 3TC (middle panel) or ABC (lower panel) treatment *in vivo* **(B)** The current-spike relationships indicate that there was no significant difference in firing among mPFC neurons from 3TC, ABC or SAL-pretreated rats (SAL vs. 3TC: n = 9 vs. 12. 3TC effect: *F*_(1,19)_ = 0.009, *p* = 0.925; current effect: *F*_(11,209)_ = 309.6, *p*<0.001; interaction: *F*_(11,206)_ = 0.502, *p* = 0.901; SAL vs. ABC: n = 9 vs. 12. ABC effect: *F*_(1,19)_ = 1.136, *p* = 0.300; current effect: *F*_(11,209)_ = 211.4, *p*<0.001; interaction: *F*_(11,206)_ = 2.557, *p* = 0.005). **(C)** Sample traces showing the firing in response to *V*_m_ hyperpolarization in mPFC neurons following 4 wks pretreatment of 70% DMSO (the solvent for DTG, upper panel), or DTG (lower panel). **(D)** The current-spike relationships indicate that there was no significant difference in firing between neurons from 4 wks DTG-pretreated and 4 wks DMSO-pretreated rats (n = 14/ea. DTG effect: *F*_(1,26)_ = 0.587, *p* = 0.450; current effect: *F*_(11,286)_ = 308.1, *p*<0.001; interaction: *F*_(11,286)_ = 1.04, *p* = 0.411).

Triumeq is co-formulated by three individual ARVs, including 3TC, ABC, and DTG. To identify the influence of each ARV alone on mPFC neuronal activity, we further assessed firing of mPFC pyramidal neurons in rats that have received daily s. c. pretreatment of either 3TC, ABC, or DTG respectively for 4 wks. We found that none of the 3 individual ARVs was able to significantly alter firing of mPFC neurons (Figures 8A–D). These results differ from that after 4 wks chronic treatment of Triumeq, suggesting that there may be additive or interactive effects among these ARVs in affecting mPFC neuronal excitability. Alternatively, longer period of treatment time may be needed for the individual ARVs to induce hyperexcitability among mPFC neurons.

## Discussion

Our study indicates that Triumeq (a first-line cART regimen for treating HIV/AIDS) alters the functional activity of mPFC pyramidal neurons, and it does so through increasing Ca^2+^ influx via overactivation/overexpression of VGCCs, including, but may not be limited to, LVA-Cav1.3 L-channels. Specifically, exposure to Triumeq, either acutely *in vitro* or chronically *in vivo*, increased firing of mPFC neurons in a dose- and/or time-dependent manner. This Triumeq-induced neuronal hyperexcitability is mediated by Ca^2+^ dysregulation resulting from VGCC overactivation and increased mRNA expression of LVA Cav1.3 L-channels. Acute exposure to lamivudine (a.k.a. 3TC), one of the three ARVs that co-formulate Triumeq, also increased firing and Ca^2+^ influx via VGCCs in hyperactive mPFC pyramidal neurons. In contrast, neither a subchronic (2 wks) treatment of Triumeq *in vivo*, nor chronic (4 wks) treatment of individual ARVs that co-formulate Triumeq *in vivo*, significantly alters mPFC neuronal activity. Collectively, these novel findings indicate that chronic Triumeq *in vivo* abnormally increases excitability of mPFC pyramidal neurons, which is mediated by overactivation and/or overexpression of VGCCs, including, but may not limited to, L-type Ca^2+^ channels.

We demonstrated that Triumeq-induced increase of mPFC neuronal firing is dosage- and time-dependent, which could have bi-directional, therapeutic and/or neurotoxic consequences, respectively. For instance, neither the lowest concentrations of acute Triumeq *in vitro* (which were equivalent to that found in the CSFs of PLWH on these ARVs) (Calcagno et al., 2014), nor a subchronic treatment (2 wks) of Triumeq *in vivo*, instantaneously affected firing of mPFC neurons. However, higher concentrations of acute Triumeq, or chronic treatment (4 wks) of this cART *in vivo*, facilitated mPFC neuronal activity. These findings suggest that an acute or subchronic clinical dosage of Triumeq is well-tolerated by glutamatergic mPFC pyramidal neurons, which allow them to maintain normal functional activity. Nevertheless, neuronal activity could be increased by raising the dosages (reflected by higher concentrations) of Triumeq in the brain, or prolonging the period of this particular cART. Therefore, it is likely that under the circumstances in which the mPFC function begins to be diminished by aging (Chen et al., 2019), or by HIV-induced overactivation that ultimately leads to inactivation of some mPFC neurons (Wayman et al., 2012; Napier et al., 2014; Wayman et al., 2015; Khodr et al., 2016; Wayman et al., 2016; Khodr et al., 2018), such an increased neuronal activity may exert a positive role in maintaining normal function of the mPFC, or delaying progression of HAND. Both may contribute to the therapeutic effects of Triumeq, in addition to suppressing HIV replication.

However, chronic Triumeq exposure to the brain could gradually and persistently increase the excitability of mPFC neurons, rendering them more susceptible and vulnerable to deleterious excitatory stimuli in the CNS HIV reservoirs. Our previous studies demonstrate that in the context of neuroHIV, in which HIV-1 and neuroinflammation induce neuronal hyperactivity, persistent neuronal hyperexcitability drives mPFC neurons from overactivation to inactivation (Wayman et al., 2012; Napier et al., 2014; Wayman et al., 2015; Khodr et al., 2016; Wayman et al., 2016; Khodr et al., 2018; Chen et al., 2019); and such dysregulation is exacerbated by psychostimulants, which also induce hyperactivity. Under these comorbid conditions, chronic Triumeq-induced neuronal hyperactivity could enhance HIV-induced neurotoxicity by further disturbing neuronal Ca^2+^ dysregulation (see below); and that may ultimately contribute to the underlying mechanism of HAND. In addition, our study also suggests a dysfunction of voltage-gated sodium channels (VGSCs, reflected by reduced firing threshold) in these hyperactive mPFC neurons. In combination with Triumeq-induced VGCC overactivation, such a reduction in firing threshold could further promote neuronal hyperactivity. On the other side, our findings also suggest a self-protective mechanism, by which the acute effects of individual ARVs (that co-formulate Triumeq) on facilitating firing were restricted, and normal activity was maintained in response to chronic treatment of each ARV alone. This potential mechanism may work during a subchronic treatment of Triumeq. Alternatively, they also suggest additive or interactive effects of these ARVs on inducing neuronal hyperactivity following persistent treatment *in vivo*.

Another important finding of the present study is that Triumeq-induced mPFC neuronal hyperexcitability is associated with a significant increase in Ca^2+^ influx through overactive VGCCs. This Ca^2+^ dysregulation was found both *in vitro* in response to acute Triumeq exposure, and *in vivo* following chronic Triumeq treatment. Similar to its effects on firing, a lower concentration of Triumeq *in vitro*, which was equivalent to the drug level found in the CSF of PLWH on this cART (Calcagno et al., 2014) and described here as 1-fold (1×), did not alter Ca^2+^ spikes. But higher concentrations (10× or 100× greater than a clinical dosage) significantly increases Ca^2+^ influx via VGCCs. Equal importantly, the increased firing was associated with enhanced Ca^2+^ influx via overactive VGCCs. Moreover, the widened action potentials and reduced rheobase found in hyperactive mPFC neurons were also in agreement with this increased Ca^2+^ influx. Together, these results not only indicate that a regular clinical dosage of Triumeq, either given acutely or for a relatively short period of time, does not induce neuronal hyperactivity and overactivation of VGCCs, but also suggest that mPFC neurons are able to integratively contain Triumeq-induced hyperexcitability by keeping neuronal Ca^2+^ homeostasis under control, though only for a short period of time.

Similar changes in neuronal Ca^2+^ homeostasis was also induced by lower concentrations of 3TC (1–4 µM) *in vitro*, which significantly enhanced Ca^2+^ influx via overactive VGCCs. Interestingly, at concentrations that were comparable to that found in CSF of PLWH receiving this ARV (Johnson et al., 1999), 3TC was *unable* to increase firing, either acutely *in vitro* or chronically *in vivo*. This finding suggests that although a regular clinical dosage of 3TC promotes VGCC activity, it is incapable to cause neuronal hyperactivity. However, higher concentrations of, persistent exposure to, or combined treatment of other ARVs with this ARV, could drive mPFC neurons to hyperactivity mediated by deteriorated neuronal Ca^2+^ dysregulation.

Cumulative evidence suggests that neuronal Ca^2+^ dysregulation (excessive [Ca^2+^]_in_) plays a critical role in neurotoxicity induced by ARVs (Robertson et al., 2012; Romo et al., 2012; Akay et al., 2014; Apostolova et al., 2015; Underwood et al., 2015; Vivithanaporn et al., 2016). Ironically, such neuronal Ca^2+^ dysregulation is similar to that found in the context of neuroAIDS; both are mediated by dysfunction of VGCCs (Hu, 2016), in addition to NMDARs (Haughey and Mattson, 2002; Gonzalez-Scarano and Martin-Garcia, 2005; Mattson et al., 2005). VGCCs include several different subtypes, including L-type of Ca^2+^ channels that are involved in Ca^2+^-induced signaling processes and gene expression in neurons (Ikeda, 2001; Brini et al., 2014). Our previous studies demonstrate that overactivation and overexpression of L-channels contributes to abnormal hyperactivity of mPFC pyramidal neurons in the context of neuroHIV (Khodr et al., 2016; Khodr et al., 2018; Napier et al., 2014; Wayman et al., 2015; 2016; 2012). There are two subtypes of L-channels expressed in the brain, include a HVA-Ca_v_1.2 (Cacna1c) L-channel and a LVA-Ca_v_1.3 (Cacna1d) L-channel (Lipscombe, 2002). Our previous studies suggest that overactivation and overexpression of both HVA-Ca^2+^ and LVA-Ca^2+^ channels mediates mPFC neuronal hyperactivity in the context of neuroHIV and aging (Wayman et al., 2012; Napier et al., 2014; Wayman et al., 2015; Khodr et al., 2016; Wayman et al., 2016; Khodr et al., 2018). The present study further reveals the involvement of increased mRNA expression of LVA-Ca_v_1.3 L-channels following chronic Triumeq treatment. Together, these findings indicate that, even at regular clinical dosages, persistent exposure to Triumeq *in vivo*, or to any other chronic cART regimen which is likely a common situation for PLWH, may eventually complicate HIV-induced neuronal hyperactivity by targeting L-channels to exacerbate neuronal Ca^2+^ dysregulation in the brain of PLWH.

However, even though many ARVs induce neurotoxicity (Kranick and Nath, 2012; Manji et al., 2013; Underwood et al., 2015), cART is still absolutely needed for treating HIV/AIDS. Thus, how to minimize the site effects of ARVs should receive a thoughtful consideration. To avoid or minimize cART-induced neurotoxicity, a better understanding of ARVs' site effects is undeniably required. The present study, in combination with previous studies of others, indicates that the responses of pyramidal neurons in the mPFC to Triumeq differ considerably from those in the hippocampal (Hipp) CA1 region to efavirenz, a non-NRTI, nNRTI (Ciavatta et al., 2017). The CSF levels of efavirenz found in PLWH taking this ARV are in the range of 23–450 nM (Tashima et al., 1999; Yilmaz et al., 2012; Aouri et al., 2016; Ciavatta et al., 2017), depending upon the condition of studies (e.g., the time point for sample collection). In contrast to the effects of Triumeq (10× or 100×) on inducing initial *increase* of neuronal activity, efavirenz (20µM, ∼40× higher than its CSF level) significantly *suppresses* firing and causes severer dendritic injury in Hipp pyramidal neurons (Ciavatta et al., 2017). Together, these findings suggest that certain ARV(s), like efavirenz, is more toxic than other ARVs (Kranick and Nath, 2012). Our findings are in agreement with this proposition. In fact, Atripla, another cART regimen co-formulated by efavirenz, 3TC, and tenofovir disoproxiln fumarate, is reported to have significantly greater site effects and less efficacy than Triumeq in treating HIV/AIDS (Walmsley et al., 2013); and therefore, has no longer been recommended as a first-line cART regimen for treating HIV/AIDS. In a parallel study, we also examined acute effects of 1× Atripla on mPFC pyramidal neurons; and found a significant increase in firing (unpublished data). This data also suggests a greater side effect of Atripla compared to Triumeq (e.g., causing mPFC neuronal hyperactivity by a low clinical dosage). However, given that almost all ARVs have a similar site effect in inducing neurotoxicity, especially with higher dosages and/or after persistent treatment either *in vitro* or *in vivo*, the present study will not exclude the likelihood that enduring Triumeq treatment could also induce neuronal dysfunction and injury in the key brain regions that regulate neurocognition. Thus, out study also suggests that caution should be taken seriously to raise levels of ARVs in the brain of PLWH.

In summary, the present study demonstrates that chronic Triumeq treatment *in vivo* induces mPFC neuronal hyperactivity, which is due partly to neuronal Ca^2+^ dysregulation mediated by overactivation/overexpression of VGCCs, including, but not limited to, LVA-Ca_v_1.3 L-channels. Our novel findings also suggest that, while cART effectively inhibits HIV-1 replication, chronic exposure to Triumeq *in vivo* could complicate HIV-induced neurotoxicity due to abnormal increase of Ca^2+^ influx and excessive [Ca^2+^]_in_. Therefore, there may be an increasing risk for PLWH who are on chronic cART to eventually experience an undeserved impact from the site effects of ARVs on neurons in the brain region(s) that regulate neurocognition; and that may contribute to the underlying mechanism of HAND. Nevertheless, because cART is unreplaceable for treating HIV/AIDS, such side effects of ARVs will not restrict cART for PLWH. Understanding this reality and elucidating the mechanism underlying ARVs-induced neurotoxicity should ultimately help us to develop new and more effective therapeutic strategies to combating against HIV/AIDS, while minimizing the side effects of cART.

## Data Availability Statement

The raw data supporting the conclusions of this article will be made available by the authors, without undue reservation.

## Ethics Statement

The animal study was reviewed and approved by the IACUC of Rush University Medical Center.

## Author Contributions

LC and X-TH conceived and designed the study. LC performed experiments, analyzed data and prepared the original manuscript draft. LC, LA-H, and XH reviewed and edited the manuscript. LA-H and XH acquired funding to perform the projects.

## Abbreviations

PLWH, People living with HIV; HAND, HIV-associated neurocognitive disorders; cART, combined antiretroviral therapy; ARVs, antiretroviral drugs; mPFC, medial prefrontal cortex; VGCC, voltage-gated Ca2+ channel; WHO, World Health Organization; 3TC, lamivudine; ABC, abcavir; DTG, dolutegravir; NRTI, nucleotide reverse transcriptase inhibitor; INSTI, integrase strand transfer inhibitor; wk, week; RMP, resting membrane potential; AP, action potential; ×, fold; rm, repeated measures; L-channels, L-type VGCCs; LVA, low voltage-activated; HVA, high voltage-activated; Hipp, hippocampal; nNRTI, non-NRTI.

## Funding

This work was supported by the National Institutes of Health (NIH) grants number: NS084817, DA044552 and DA044552-03S1 (X-TH), DA033966, MH122241, and NS060632 (LA-H).

## Conflict of Interest

The authors declare that the research was conducted in the absence of any commercial or financial relationships that could be construed as a potential conflict of interest.
